# Parvovirus B19 in Croatia: A Large-Scale Seroprevalence Study

**DOI:** 10.3390/medicina57111279

**Published:** 2021-11-21

**Authors:** Tatjana Vilibic-Cavlek, Irena Tabain, Branko Kolaric, Klara Mihulja, Lana Blazevic, Maja Bogdanic, Dan Navolan, Natasa Beader, Anna Mrzljak

**Affiliations:** 1 Department of Virology, Croatian Institute of Public Health, 10000 Zagreb, Croatia; irena.tabain@hzjz.hr (I.T.); maja.bogdanic11@gmail.com (M.B.); 2 School of Medicine, University of Zagreb, 10000 Zagreb, Croatia; natasaeli@gmail.com (N.B.); anna.mrzljak@gmail.com (A.M.); 3 Department of Public Health Gerontology, Andrija Stampar Teaching Institute of Public Health, 10000 Zagreb, Croatia; branko.kolaric@stampar.hr; 4 Department of Epidemiology, Faculty of Medicine, University of Rijeka, 51000 Rijeka, Croatia; 5 Zagreb County Family Medicine Division, 10380 Sveti Ivan Zelina, Croatia; kmihulja@gmail.com; 6 Department of Epidemiology, Croatian Institute of Public Health, 10000 Zagreb, Croatia; lana.blazevic@hzjz.hr; 7 Department of Obstetrics-Gynecology, “Victor Babes” University of Medicine and Pharmacy, 300041 Timisoara, Romania; navolan@umft.ro; 8 Department of Clinical and Molecular Microbiology, University Hospital Center, 10000 Zagreb, Croatia; 9 Department of Gastroenterology and Hepatology, University Hospital Centre Zagreb, 10000 Zagreb, Croatia

**Keywords:** parvovirus B19, seroprevalence, Croatia, pregnancy, hemodialysis, transplant

## Abstract

*Background and Objectives*: Seroepidemiological studies indicate that parvovirus B19 circulates in all areas of the world, although with some differences. The aim of this study is to analyze the seroprevalence of parvovirus B19 in the Croatian population. *Materials and Methods*: From 2010 to 2021, 1538 serum samples from different populations were tested for the presence of parvovirus B19 IgM/IgG antibodies. Serological tests were performed using a commercial enzyme-linked immunosorbent assay. *Results*: IgG antibodies were detected in 986/64.1% of participants with differences (*p* < 0.001) among the following population groups: 42.4% of children and adolescents, 67.1% of the adult general population, 66.7% of hemodialysis patients, and 65.6% of liver transplant recipients. Seroprevalence increased with age, from 30.0% in the 6 months–9 years age group to 69.0% in the 40–49 years age group, and remained stable thereafter (68.8–73.3%). There was no difference in the seropositivity among males (66.1%) and females (63.1%), as well as the place of residence (suburban/rural 63.9%, urban 64.1%). IgM antibodies (current/recent infection) were found in 61/4.0% of participants with the highest seropositivity in the youngest age group (11.1%). In pregnant women, seroprevalence was higher in women with an unfavorable obstetric history compared with a normal pregnancy (IgG 71.0% vs. 62.6%; IgM 6.5% vs. 2.4%), but these differences were not significant. Logistic regression showed that the adult population had almost three times higher risk of IgG seropositivity compared to children/adolescents (general population OR = 2.777, 95% CI = 2.023–3.812; hemodialysis patients OR = 2.586, 95% CI = 1.531–4.367; and transplant patients OR = 2.717, 95% CI = 1.604–4.603). A one-year increase in age increased the risk of IgG seroprevalence (OR = 1.017; 95% CI = 1.011–1.022). *Conclusions*: Older age was the main risk factor for IgG seropositivity. Hemodialysis and organ transplantation seem unrelated to the increased parvovirus B19 seroprevalence. The role of parvovirus B19 in the etiology of TORCH infections needs to be studied further.

## 1. Introduction

Parvovirus B19 is a common viral pathogen with a worldwide distribution. It is a small, non-enveloped virus with a linear, single-stranded DNA genome that belongs to the *Erythroparvovirus* genus of the family *Parvoviridae*. The capsid consists of 60 viral proteins (VP): VP2 constitutes about 95% of the viral capsid proteins, while VP1 constitutes about 5%. The primary receptor for parvovirus B19 is globoside or the P antigen, while various other co-receptors are presumed to be involved in the viral entry. The replication takes place in the nucleus of the infected cells. Parvovirus B19 mainly infects human erythroid progenitor cells; however, recent clinical studies indicate that the virus also infects nonerythroid lineage cells and can be associated with different disease outcomes [1].

Seroprevalence studies indicate that the virus circulates in all areas of the world, although with some differences. Seasonal outbreaks occur every 3–5 years. In the general adult population, seroprevalence rates vary from 29% to 72% [2,3,4,5,6]. Most individuals with parvovirus B19 infections are asymptomatic or have mild, non-specific symptoms. However, different clinical conditions are associated with the infection, such as erythema infectiosum; arthropathy; transient aplastic crisis; chronic red cell aplasia; and papular, purpuric eruptions on the hands and feet (“gloves and socks” syndrome) [7,8].

In childhood, parvovirus B19 causes erythema infectiosum (“fifth disease”), a febrile disease with a rash, while, in adults, infection is more commonly presented as a non-specific febrile illness. Arthralgia is more common in adolescents and adults, affecting up to 60% of persons [7]. In addition, parvovirus B19 may be responsible for transient aplastic crises in patients with sickle cell anemia and spherocytosis, as well as for prolonged anemia in immunocompromised people [9,10]. Furthermore, anemia was reported in 99% of solid-organ transplant recipients with the parvovirus B19 infection. Hepatitis, myocarditis, and pneumonitis were also reported in association with parvovirus B19 in immunocompromised populations [11,12]. Furthermore, parvovirus B19 infections associated with acute myocarditis leading to heart failure has been addressed in a single case report [13].

Acute parvovirus B19 infection in pregnant women is an important cause of fetal morbidity. The estimated risk of transplacental infection is 17–33% [14]. There does not appear to be any evidence that the parvovirus B19 infection increases the risk of congenital anomalies; however, fetal anemia and hydrops are reported after maternal infection during pregnancy [15].

In Croatia, there are no data on the prevalence of parvovirus B19. The aim of the present study was to analyze the seroprevalence and possible risk factors for parvovirus B19 infection in different population groups.

## 2. Materials and Methods

During an 11-year period (2010–2021), a total of 1538 consecutive serum samples from Croatian residents were tested for the presence of parvovirus B19 IgM and IgG antibodies at the Croatian Institute of Public Health, the largest public health institute in the country. In the tested group, there were 502 (32.7%) males and 1036 (67.3%) females aged from 1 month to 87 years. The study included different population groups: children and adolescents (<18 years; N = 184), the general adult population (>18 years; N = 1174), patients on hemodialysis (N = 90), and liver transplant recipients (N = 90). Pregnant women (N = 304) were further separated in a subgroup with normal pregnancy (N = 211) and unfavorable obstetric history (previous spontaneous abortions, infertility, and sterility) (N = 93).

Serologic tests were performed using a commercial enzyme-linked immunosorbent assay (ELISA; Euroimmun, Lübeck, Germany). The results were interpreted according to the manufacturer’s recommendations, as follows: IgM ratio < 0.8 negative; 8.8–1.1 borderline; >1.1 positive; IgG RU/mL < 4 negative; 4–5.5 borderline and >5.5 positive.

### Statistical Analysis

The frequencies are presented with 95% confidence intervals (CI). The differences between groups were tested using Fischer’s exact test. The strength of the association between dependent and independent variables was assessed using logistic regression. Due to mother-to-child passive immunity, the youngest age group (<6 months) was excluded from the logistic regression model. For statistical analysis, the software package STATA/IC ver 11.2 (StataCorp LP, College Station, TX, USA) was used. The level of statistical significance was α = 0.05.

## 3. Results

### 3.1. Parvovirus B19 IgM and IgG Testing

The results of serology testing are presented in Table 1 and Table 2.

Parvovirus B19 IgG antibodies were detected in 986 (64.1%) participants. The seropositivity differed significantly (*p* < 0.001) among population groups: children and adolescents (42.4%); the general adult population (67.1%); hemodialysis patients (66.7%); and liver transplant recipients (65.6%). According to age, 65.2% of children less than 6 months old were parvovirus B19 IgG seropositive, due to transplacentally derived maternal antibodies. IgG seroprevalence increased steadily, from 30.0% in the 6 months–9 years age group to 69.0% in the 40–49 years age group, and remained stable thereafter (68.8–73.3%). There was no difference in the seropositivity among males and females (66.1% and 63.1%, respectively). In addition, no difference was observed among residents in suburban/rural areas (63.9%) and residents in urban areas (64.1%).

Parvovirus B19 IgM antibodies (current/recent infection) were found in 61 (4.0%) participants (Table 2). Acute infections were detected in all age groups; however, the highest IgM seropositivity was found in the age group > 6 months–9 years (11.1%), with a continuous decline with age to 1.2% in the age group 50–59 years and older participants (*p* < 0.001). The prevalence of acute infections did not differ between males (4.3%) and females (3.2%).

In a group of pregnant women, both IgG (71.0% vs. 62.6%) and IgM (6.5% vs. 2.4%) seroprevalence was higher in women with an unfavorable obstetric history compared with normal pregnancy, but these differences were not statistically significant (IgG *p* = 0.192; IgM *p* = 0.098).

### 3.2. Risk Analysis for Parvovirus B19 Seropositivity

The results of logistic regression (Table 3) showed that the adult population had almost 3 times higher risk of IgG seropositivity (general population OR = 2.777, 95% CI = 2.023–3.812; hemodialysis patients OR = 2.586, 95% CI = 1.531–4.367; transplant patients OR = 2.717, 95% CI = 1.604–4.603) compared to children and adolescents. A 1-year increase in age increased the risk of IgG seroprevalence (OR = 1.017; 95% CI = 1.011–1.022). Gender and the area of residence were not associated with parvovirus B19 IgG seroprevalence.

Compared to children and adolescents, adults were less likely to be IgM positive (acute infections): the adult general population OR = 0.342, 95% CI = 0.192–0.607; hemodialysis patients OR = 0.104, 95% CI = 0.013–0.798. In addition, residents of urban areas showed a lower risk of IgM positivity compared to residents of suburban/rural areas (OR = 0.509, 95% CI = 0.285–0.907) (Table 4).

## 4. Discussion

In this study, which is the first seroepidemiological study on parvovirus B19 in Croatia, the overall seroprevalence was 64.1%. Similar seroprevalence rates were reported in the Netherlands (61%) [16], Israel (61.4%) [4], Brazil (62.8%) [17], and Germany, in the Frankfurt am Main area (62.9%) [18]. Higher seropositivity (72.1%) was found in the German National Health Survey conducted in former Eastern and Western Germany [5] and in Iran (Tehran City, 86.6%) [19]. Lower seroprevalence rates were documented in the general Polish population (52.9%) [20] as well as in England and Wales (56.3%) [21], while a very low seroprevalence was detected both in children and adults (20.7% and 36%, respectively) in Central Anatolia, Turkey [2].

Parvovirus B19 IgG seroprevalence increased significantly with age in the Croatian cohort population, with 52.2% seropositive persons at the age of 19 years. Gradually rising seroprevalence rates of up to 69.0% were observed in 40–49-year-olds; subsequently, seroprevalence rates remained stable (69.1–73.3%). A similar age-related seroprevalence was found in Poland. The first peak of seropositive persons was observed in pre-school children with the proportion of seropositive patients achieving 65.5% among 15–19-year-olds and 81.2% among 40–49-year-olds [20]. In addition, a gradual increase in the seroprevalence was observed in the German population, with seropositivity of 71% in adults aged from 20 to >60 years [18]. In England and Wales, seroprevalence increased nonlinearly with age [21] whereas, in the Netherlands, no positive correlation with increasing age, nor significant differences between age groups, were found in the urban adult Amsterdam population [16]. In countries with a high overall seropositivity, such as Iran, 79.3% of 5–9-year-old children showed IgG antibodies with an increase to 93.5% in young adults (20–25 years), indicating the earlier transmission of parvovirus B19 [19]. The IgG seropositivity of 65.2% found in the sera of newborn infants in this study reflects the maternal antibody status.

In this study, no difference was found in the IgG seropositivity between males (66.1%) and females (63.1%). No gender differences in the parvovirus B19 seroprevalence rates were found in several other studies (Turkey, Israel, and Poland) [2,4,20]. However, significantly higher seropositivity in women compared to men (42.0% vs. 36.2%) was found in another Turkish study [22]. Similarly, in a German study, differences were observed between females (73.3%) and males (70.9%) [5].

Some studies showed difference in the parvovirus B19 seroprevalence, according to the area of residence. A higher level of seropositivity among residents in urban areas compared to rural areas was found in Iran (88% vs. 84.3%) [19]. In contrast, in Germany, the inhabitants of small cities were more often seropositive (74.8%) than the inhabitants of big cities (69.0%) [5]. Our study found no differences in the IgG seroprevalence regarding the area of residence (suburban/rural areas 63.9%; urban areas 64.1%).

Few studies analyzed the seroprevalence of parvovirus B19 in hemodialysis patients, mainly from the Middle East. Chronic anemia is a common problem in hemodialysis patients that require blood transfusion. Our results found no difference in the seropositivity in hemodialysis patients (65.6%) compared to the general adult population (67.1%), suggesting no increased risk of parvovirus B19 infection regarding receiving blood transfusions. In Iran and Brazil, the prevalence of parvovirus B19 IgG antibodies among hemodialysis patients was 54% and 67.5%, respectively [23,24], while, in Iraq, a high seroprevalence of 90.2% was observed [25].

In general, the recipients of solid organs or hematopoietic stem cells are at an increased risk of viral reactivation originating from previous latent or persisting viral infections or pathogens transmitted by the transplant. However, the parvovirus B19 seroprevalence of liver transplant recipients (66.7%) did not differ from other adult populations in our study, and none of the tested patients had an acute infection. Seroprevalence studies in the transplant population are scarce, but similar seroprevalence rates were also confirmed in Iranian kidney transplant recipients (69.2%) who suffered from anemia [26]. A German group investigated 371 adult transplant recipients (kidney, liver, heart, and bone marrow), showing that seroprevalence rates before and after transplants are almost identical (82% and 83%, respectively) [27], which is in line with our study.

With a reduced incidence, acute infections can occur in adults. In this study, acute parvovirus B19 infections were the most frequent in the youngest age group (11.1%) and adolescents (8.9%). However, 5.5% and 4.9% of acute infections were also reported in the age group of 20–39 years, which could be explained by the close contact with small children because of family circumstances, which probably increase the risk of virus transmission.

Data on the seroprevalence of parvovirus B19 infection in pregnant women are important for identifying seronegative women who are at risk of primary infection during pregnancy. Seroprevalence studies in European countries showed seroprevalence rates in pregnant women ranging from 55% to 74% [19,20,28,29,30,31,32]. Furthermore, a recently published study conducted among childbearing-aged women residing in Brazil, Mexico, Germany, Poland, Turkey, and China revealed a very low seropositivity in Chinese women (7.5%) compared to the other countries (30–65%) [33]. The results of this study showed that the seroprevalence in pregnant women (65.5%) was similar to that of the general population (64.1%). Although women with an unfavorable obstetric history had higher seropositivity (71.0%), compared with women who had a normal pregnancy (62.6%), the difference was not significant. Similarly, there was no difference in the IgM seroprevalence rates between these groups (6.5% vs. 2.4%). Similar to our results, a study on the seroprevalence of parvovirus B19 in St. Petersburg found no difference in the IgG seropositivity among pregnant women and women with recurrent abortions (75.3% and 66.9%, respectively) [34]. However, in a study from Egypt, a significantly higher IgM seroprevalence rate of 84% was found in women with recurrent abortions compared to 20% in the control group, suggesting the possible role of parvovirus B19 in recurrent abortions [35]. Additionally, the frequency of abortions in parvovirus B19 IgG seropositive women was showed to be 4 times higher than in the seronegative group of Iranian pregnant women [36].

The limitation of this study, which needs to be stressed, is that consecutive serum samples were tested; therefore, people tested were not necessarily representative of that area population.

## 5. Conclusions

The results of this first large seroepidemiological study demonstrates that 64.1% of the Croatian population shows the exposure to parvovirus B19, with (increasing) age as the main risk factor for IgG seropositivity. Gender specific differences in the seropositivity were not observed. Hemodialysis patients and transplant recipients were not found to be at an increased risk for the parvovirus B19 infection. The role of parvovirus B19 as a TORCH agent needs to be studied further.

## Figures and Tables

**Table 1:** Prevalence of parvovirus B19 IgG antibodies.

Characteristic		N Tested (%)	N IgG Positive (%)	95% CI	*p*
Population group	Children and adolescents (<18 years)	184 (12.0)	78 (42.2)	35.2–49.9	< 0.001
	Adult general population	1175 (76.4)	789 (67.1)	64.4–69.8	
	Hemodialysis patients	90 (5.8)	59 (65.6)	54.8–75.3	
	Transplant patients	90 (5.8)	60 (66.7)	55.9–76.3	
Gender	Male	503 (32.7)	332 (66.1)	61.7–70.1	0.282
	Female	1036 (67.3)	654 (63.1)	60.1–66.1	
Age group	<6 months	23 (1.5)	15 (65.2)	42.7–83.6	< 0.001
	≥6 months–9 years	90 (5.9)	27 (30.0)	20.8–40.6	
	10–19 years	90 (5.9)	47 (52.2)	41.4–62.9	
	20–29 years	222 (14.4)	140 (63.1)	56.3–69.4	
	30–39 years	410 (26.6)	265 (64.6)	59.8–69.3	
	40–49 years	226 (14.7)	156 (69.0)	62.6–75.0	
	50–59 years	246 (16.0)	170 (69.1)	62.6–74.5	
	60–69 years	146 (9.5)	107 (73.3)	65.3–80.3	
	70 + years	85 (5.5)	59 (69.4)	58.4–79.0	
Area of residence	Urban	1272 (64.1)	816 (64.1)	61.4–66.8	0.944
	Suburban/rural	266 (35.9)	170 (63.9)	57.8–69.7	
Pregnant women	Normal pregnancy	211 (69.4)	132 (62.6)	55.7–69.1	0.192
	Unfavorable obstetric history	93 (30.6)	66 (71.0)	60.6–79.9	
All participants		1538	986 (64.1)	61.6–66.5	

**Table 2:** Prevalence of parvovirus B19 IgM antibodies.

Characteristic		N Tested (%)	N IgM Positive (%)	95% CI	*p*
Population group	Children and adolescents (<18 years)	184 (12.0)	18 (9.8)	5.9–15.0	<0.001
	Adult general population	1175 (76.4)	42 (3.6)	2.6–4.8	
	Hemodialysis patients	90 (5.8)	1 (1.1)	0.1–6.1	
	Transplant patients	90 (5.8)	0 (0.0)	0.0–4.0 *	
Gender	Male	503 (32.7)	45 (4.3)	3.2–5.8	0.330
	Female	1036 (67.3)	16 (3.2)	1.8–5.1	
Age group	<6 months	23 (1.5)	0 (0.0)	0.0–14.8 *	<0.001
	≥6 months–9 years	90 (5.9)	10 (11.1)	5.5–19.5	
	10–19 years	90 (5.9)	8 (8.9)	3.9–16.8	
	20–29 years	222 (14.4)	12 (5.4)	2.8–9.3	
	30–39 years	410 (26.6)	20 (4.9)	3.0–7.4	
	40–49 years	226 (14.7)	7 (3.1)	1.3–6.3	
	50–59 years	246 (16.0)	3 (1.2)	0.3–3.5	
	60–69 years	146 (9.5)	0 (0.0)	0.0–2.5 *	
	70 + years	85 (5.5)	1 (1.2)	0.1–6.4	
Area of residence	Urban	1272 (64.1)	44 (3.5)	2.5–4.6	0.036
	Suburban/rural	266 (35.9)	17 (6.4)	3.8–10.0	
Pregnant women	Normal pregnancy	211 (69.4)	5 (2.4)	0.1–5.4	0.098
	Unfavorable obstetric history	93 (30.6)	6 (6.5)	2.4–13.5	
All participants		1538	61 (4.0)	3.0–5.1	

* One-sided 97.5% confidence interval.

**Table 3:** Logistic regression risk analysis for the parvovirus B19 IgG seropositivity.

Characteristic	OR IgG	95% CI
Female (Ref.) vs. male gender	0.725	0.405–1.295
Age (one-year increase)	1.017	1.011–1.022 *
Urban (Ref.) vs. suburban/rural area of residence	1.001	0.759–1.319
Children and adolescents (<18 years)	Ref.	
Adult general population	2.777	2.023–3.812 *
Hemodialysis patients	2.586	1.531–4.367 *
Transplant patients	2.717	1.604–4.603 *
Women with unfavorable obstetric history (Ref.) vs. normal pregnancy	1.462	0.863–2.479

* Statistically significant.

**Table 4:** Logistic regression risk analysis for the parvovirus B19 IgM seropositivity.

Characteristic	OR IgM	95% CI
Female (Ref.) vs. male gender	0.725	0.405–1.295
Age (one-year increase)	0.964	0.951–0.978 *
Urban (Ref.) vs. suburban/rural area of residence	0.509	0.285–0.907 *
Children and adolescents (<18 years)	Ref.	
Adult general population	0.341	0.192–0.607 *
Hemodialysis patients	0.104	0.013–0.798 *
Transplant patients	NA	NA
Women with unfavorable obstetric history (Ref.) vs. normal pregnancy	2.841	0.844–9.997

* Statistically significant; NA = not applicable.

## Data Availability

Not applicable.

## References

[1] Ganaie S.S., Qiu J. (2018). Recent Advances in Replication and Infection of Human Parvovirus B19. Front. Cell Infect. Microbiol..

[2] Türk Dağı H., Ozdemir M., Baykan M., Baysal B. (2010). Investigation of parvovirus B19 seroprevalence in various age groups in Central Anatolia Region, Turkey. Mikrobiyol. Bulteni..

[3] Ziyaeyan M., Pourabbas B., Alborzi A., Mardaneh J. (2007). Prevalence of antibody to human parvovirus B19 in pre-school age/young adult individuals in Shiraz, Iran. Pak. J. Biol. Sci..

[4] Mor O., Ofir I., Pavel R., Bassal R., Kra-Oz Z., Cohen D., Shohat T., Mendelson E. (2016). Parvovirus B19V infection in Israel: Prevalence and occurrence of acute infection between 2008 and 2013. Epidemiol. Infect..

[5] Röhrer C., Gärtner B., Sauerbrei A., Böhm S., Hottenträger B., Raab U., Thierfelder W., Wutzler P., Modrow S. (2008). Seroprevalence of parvovirus B19 in the German population. Epidemiol. Infect..

[6] Faddy H.M., Gorman E.C., Hoad V.C., Frentiu F.D., Tozer S., Flower R.L.P. (2018). Seroprevalence of antibodies to primate erythroparvovirus 1 (B19V) in Australia. BMC Infect. Dis..

[7] Servey J.T., Reamy B.V., Hodge J. (2007). Clinical presentations of parvovirus B19 infection. Am. Fam. Physic..

[8] Smith S.B., Libow L.F., Elston D.M., Bernert R.A., Warschaw K.E. (2002). Gloves and socks syndrome: Early and late histopathologic features. J. Am. Acad. Dermatol..

[9] Slavov S.N., Kashima S., Pinto A.C., Covas D.T. (2011). Human parvovirus B19: General considerations and impact on patients with sickle-cell disease and thalassemia and on blood transfusions. FEMS Immunol. Med. Microbiol..

[10] Mrzljak A., Kardum-Skelin I., Cvrlje V.C., Kanizaj T.F., Sustercic D., Gustin D., Kocman B. (2010). Parvovirus B 19 (PVB19) induced pure red cell aplasia (PRCA) in immunocompromised patient after liver transplantation. Coll. Antropol..

[11] Eid A.J., Brown R.A., Patel R., Razonable R.R. (2006). Parvovirus B19 infection after transplantation: A review of 98 cases. Clin. Infect. Dis..

[12] Mrzljak A., Tabain I., Premac H., Bogdanic M., Barbic L., Savic V., Stevanovic V., Jelic A., Mikulic D., Vilibic-Cavlek T. (2019). The role of emerging and neglected viruses in the etiology of hepatitis. Curr. Infect. Dis. Rep..

[13] Bock C.T., Klingel K., Kandolf R. (2010). Human parvovirus B19-associated myocarditis. N. Engl. J. Med..

[14] Lamont R.F., Sobel J.D., Vaisbuch E., Kusanovic J.P., Mazaki-Tovi S., Kim S.K., Uldbjerg N., Romero R. (2011). Parvovirus B19 infection in human pregnancy. BJOG.

[15] Ornoy A., Ergaz Z. (2017). Parvovirus B19 infection during pregnancy and risks to the fetus. Birth Defects Res..

[16] van Rijckevorsel G.G., Sonder G.J., Schim van der Loeff M.F., van den Hoek J.A. (2009). Population-based study on the seroprevalence of parvovirus B19 in Amsterdam. J. Med. Virol..

[17] Azevedo K.M., Setúbal S., Camacho L.A., Velarde L.G., Oliveira S.A. (2009). Seroepidemiological study of human parvovirus B19 among human immunodeficiency virus-infected patients in a medium-sized city in Rio de Janeiro, Brazil. Mem. Inst. Oswaldo Cruz..

[18] Reinheimer C., Allwinn R., Doerr H.W., Wittek M. (2010). Seroepidemiology of parvovirus B19 in the Frankfurt am Main area, Germany: Evaluation of risk factors. Infection.

[19] Salimi V., Gouya M., Esteghamati A., Safaie A., Heshmat R., Saadatmand Z., Mokhtari-Azad T. (2008). Seroepidemiology of human parvovirus B19 in 5–25 year old age people in Iran. Iran. J. Public Health.

[20] Siennicka J., Stefanoff P., Trzcińska A., Rosińska M., Litwińska B. (2006). Seroprevalence study of parvovirus B19 in Poland. Przegl. Epidemiol..

[21] Vyse A.J., Andrews N.J., Hesketh L.M., Pebody R. (2007). The burden of parvovirus B19 infection in women of childbearing age in England and Wales. Epidemiol. Infect..

[22] Aktaş O., Aydin H., Uslu H. (2016). Serological prevalence of human parvovirus B19 in diseases or disorders related to different human body systems. Turk J. Med. Sci..

[23] Sharif A., Aghakhani A., Velayati A.A., Banifazl M., Sharif M.R., Razeghi E., Kheirkhah D., Kazemimanesh M., Bavand A., Ramezani A. (2016). Frequency and genotype of human parvovirus B19 among Iranian hemodialysis and peritoneal dialysis patients. Intervirology.

[24] Alves M.T., Vilaça S.S., Godoi L.C., Rezende Júnior L., Carvalho M.G., de Souza Silva F., Guimarães F.L., Fernandes A.P., Dusse L.M., Gomes K.B. (2014). Parvovirus B19 (B19) and cytomegalovirus (CMV) infections and anti-erythropoietin (anti-EPO) antibodies in patients on dialysis hyporesponsive to erythropoietin therapy. Clin. Chim. Acta.

[25] Fathom S.H., Hussein A.A. (2018). Infection rate of human parvovirus B19 among hemodialysis patients in Bequeath city. IOSR J. Pharm. Biol. Sci..

[26] Khameneh Z.R., Sepehrvand N., Sohrabi V., Ghasemzadeh N. (2014). The seroprevalence of Parvovirus B19 among kidney transplant recipients: A single-center study. Saudi J. Kidney Dis. Transpl..

[27] Plentz A., Würdinger M., Kudlich M., Modrow S. (2013). Low-level DNAemia of parvovirus B19 (genotypes 1-3) in adult transplant recipients is not associated with anaemia. J. Clin. Virol..

[28] Milošević V., Jerant-Patić V., Hrnjaković-Cvjetković I., Vukmanović-Papuga M., Radovanov-Tadić J., Kovačević G. (2007). The frequency of human parvovirus B19 infections in Vojvodina. Med. Pregl..

[29] van Gessel P.H., Gaytant M.A., Vossen A.C., Galama J.M., Ursem N.T., Steegers E.A., Wildschut H.I. (2006). Incidence of parvovirus B19 infection among an unselected population of pregnant women in the Netherlands: A prospective study. Eur. J. Obstet. Gynecol. Reprod. Biol..

[30] Mossong J., Hens N., Friederichs V., Davidkin I., Broman M., Litwinska B., Siennicka J., Trzcinska A., Van Damme P., Beutels P. (2008). Parvovirus B19 infection in five European countries: Seroepidemiology, force of infection and maternal risk of infection. Epidemiol. Infect..

[31] Barlinn R., Vainio K., Samdal H.H., Nordbø S.A., Nøkleby H., Dudman S.G. (2014). Susceptibility to cytomegalovirus, parvovirus B19 and age-dependent differences in levels of rubella antibodies among pregnant women. J. Med. Virol..

[32] Alanen A., Kahala K., Vahlberg T., Koskela P., Vainionpää R. (2005). Seroprevalence, incidence of prenatal infections and reliability of maternal history of varicella zoster virus, cytomegalovirus, herpes simplex virus and parvovirus B19 infection in South-Western Finland. BJOG.

[33] Warnecke J.M., Pollmann M., Borchardt-Lohölter V., Moreira-Soto A., Kaya S., Sener A.G., Gómez-Guzmán E., Figueroa-Hernández L., Li W., Buska K. (2020). Seroprevalences of antibodies against ToRCH infectious pathogens in women of childbearing age residing in Brazil, Mexico, Germany, Poland, Turkey and China. Epidemiol. Infect..

[34] Odland J.Ø., Sergejeva I.V., Ivaneev M.D., Jensen I.P., Stray-Pedersen B. (2001). Seropositivity of cytomegalovirus, parvovirus and rubella in pregnant women and recurrent aborters in Leningrad County, Russia. Acta Obstet. Gynecol. Scand..

[35] el-Sayed Zaki M., Goda H. (2007). Relevance of parvovirus B19, herpes simplex virus 2, and cytomegalovirus virologic markers in maternal serum for diagnosis of unexplained recurrent abortions. Arch. Pathol. Lab. Med..

[36] Khameneh Z.R., Hanifian H., Barzegari R., Sepehrvand N. (2014). Human parvovirus B19 in Iranian pregnant women: A serologic survey. Indian J. Pathol. Microbiol..

